# Adjuvant Chemotherapy for Stage III Colon Cancer

**DOI:** 10.3390/cancers12092679

**Published:** 2020-09-19

**Authors:** Julien Taieb, Claire Gallois

**Affiliations:** 1 Sorbonne Paris cite, University of Paris, 75006 Paris, France; claire.gallois@aphp.fr; 2 Siric CARPEM, Assistance Publique-Hôpitaux de Paris, Department of Gastroenterology and Digestive Oncology, Hôpital Européen Georges Pompidou, 75015 Paris, France

**Keywords:** colon cancer, adjuvant chemotherapy, prognosis

## Abstract

**Simple Summary:**

In patients with stage III colon cancer, adjuvant chemotherapy with a fluoropyrimidine combined with oxaliplatin reduces the risk of recurrence and mortality, with a treatment duration that may be shortened from 6 to 3 months in certain situations allowing to limit toxicities, especially cumulative sensitive neuropathy. However, it is difficult to effectively predict the risk of recurrence individually for each patient. It is indeed necessary not to over-treat patients with potential toxicities of chemotherapy and, conversely, not to under-treat patients at high risk of recurrence, and also to find new treatment approaches for specific subgroups. Though no single biomarker have sufficient predictive value to adapt the therapeutic strategy, we have considerably improved our knowledge of these biomarkers predictive of recurrence in localized colon cancer and many trials testing their ability to guide treatment are ongoing.

**Abstract:**

In patients with stage III colon cancer (CC), adjuvant chemotherapy with the combination of oxapliplatin to a fluoropyrimidine (FOLFOX or CAPOX) is a standard of care. The duration of treatment can be reduced from 6 months to 3 months, depending on the regimen, for patients at low risk of recurrence, without loss of effectiveness and allowing a significant reduction in the risk of cumulative sensitive neuropathy. However, our capacity to identify patients that do really need this doublet adjuvant treatment remains limited. In fact, only 30% at the most will actually benefit from this adjuvant treatment, 50% of them being already cured by the surgery and 20% of them experiencing disease recurrence despite the adjuvant treatment. Thus, it is necessary to be able to better predict individually for each patient the risk of recurrence and the need for adjuvant chemotherapy together with the need of new treatment approaches for specific subgroups. Many biomarkers have been described with their own prognostic weight, without leading to any change in clinical practices for now. In this review, we will first discuss the recommendations for adjuvant chemotherapy, and then the different biomarkers described and the future perspectives for the management of stage III CC.

## 1. Introduction

Colon cancer (CC) is the third most common cancer and the second leading cause of cancer death worldwide [1]. In localized CC, lymph node invasion is a major prognostic factor, with a 5 year-overall survival (OS) of 99% and 70% for patients with stage I and II CC, respectively, versus only 45 to 65% for stage III patients [2], representing about 35% of CC at diagnosis. 

In stage III CC patients, adjuvant fluoropyrimidine-based chemotherapy is a standard of care since the 90s based on the Moertel trial [3]. In 2004, the MOSAIC trial set up the combination of oxapliplatin to a fluoropyrimidine (FOLFOX regimen) for 6 months (12 cycles) as a new standard of care for stage III CC, by improving disease-free survival (DFS) [4] but also long-term overall survival (OS) [5] with the use of this doublet chemotherapy. However, our capacity to identify patients that do really need this doublet adjuvant treatment remains limited. It is accepted that for 100 stage III CC patients receiving fluoropyrimidine + oxaliplatin, only 30 patients at the most will benefit from this adjuvant treatment, 50 patients being already cured by the surgical removal of their tumor and 20 patients experiencing disease recurrence despite the adjuvant treatment [6]. Though adjuvant therapy benefits a limited number of treated patients, all of them will be exposed to short- and long-term toxicities induced by these chemotherapeutic agents, including long lasting cumulative sensitive neuropathy induced by oxaliplatin. 

Waiting to be able to personalize adjuvant treatment and give the right therapeutic approach to the right patient, strategies aiming at reducing treatment toxicities were tested and the reduction in treatment duration from 6 to 3 months proposed by the International Duration Evaluation of Adjuvant (IDEA) international collaboration allowed to reduce significantly treatment-induced toxicities [7].

In this review, we will first discuss the recommendations for adjuvant chemotherapy in stage III CC (timing of initiation, different regimens, treatment duration, specific situations such as elderly patients or locally advanced tumors). We will then discuss the different biomarkers described in this situation and the future perspectives for the management of stage III CC.

## 2. Timing of Adjuvant Chemotherapy

A meta-analysis in stage III CC showed that delay in initiation of adjuvant chemotherapy beyond 8 weeks after curative surgery decreased OS (RR: 1.20; 95% Confidence Interval (CI) 1.15–1.26), probably also in part due to post-operative complications and a poor general condition [8]. However, patients could still benefit from adjuvant chemotherapy with a longer delay, up to 5–6 months, but the effectiveness of chemotherapy decreases with a time elapsed of 8 weeks [9,10]. Thus, chemotherapy should be initiated 6–8 weeks after curative surgery to have the greatest benefit, and has no role beyond 6 months after surgery.

## 3. Chemotherapy Regimens

The first adjuvant chemotherapy regimen to demonstrate its efficacy in localized CC compared to surgery alone was the FULV regimen: fluorouracil (370–400 mg/m^2^) plus L-folinic acid (175–200 mg or 25 mg) daily for 5 days every month for 6 cycles [11,12,13,14] with a reduction of around 15% in the risk of death at 5 years. The semimonthly LV5FU2 regimen and capecitabine (oral fluoropyrimidine) have been shown to be as effective and better tolerated than FULV [15,16]. 

Then, the addition of oxaliplatin with FOLFOX or CAPOX regimens for 6 months has been validated with an increase in 6-year DFS and OS to 73.3% and 78.5%, respectively, as compared to 67.4% and 76% for fluoropyrimidine alone in the MOSAIC trial [4], confirmed by the NSABP C-07 [17,18] and XELOXA trials [19], with similar outcomes in improving DFS. Updated 10-year OS data of the MOSAIC trial in stage III CC confirmed these results with a 10-year OS rate of 59.0% for patients treated with LV5FU2 and 67.1% for patients treated with FOLFOX (HR, 0.80; *p* = 0.016) [5]. Compared to the FOLFOX4 regimen, modified FOLFOX6 regimen (biweekly courses of oxaliplatin 85 mg/m^2^, with leucovorin 400 mg/m^2^ and fluorouracil 400 mg/m^2^ bolus, then 46-hour intravenous fluorouracil 2400 mg/m^2^) seems more convenient and cost-effective because the patient only comes once in a day hospital, with similar DFS rates in the two adjuvant phase 3 trials using FOLFOX6m: NSABPC-08 [20] and NCCTG NO147 [21] in cross-study comparisons with the MOSAIC (using FOLFOX4) [4] and NSABPC-07 (using FLOX) trials [17].

Although they are effective in the metastatic setting, FOLFIRI, IFL and FOLFOX combined to VEGF- or EGFR-targeted therapies, did not improved outcomes in stage III CC [21,22,23,24,25,26,27]. Interestingly, we showed in the PETACC-8 trial that in the subgroup of patients with full RAS and BRAF wild-type tumors, there was a trend to a better DFS when cetuximab was added to FOLFOX with a clinically relevant adjusted HR of 0.76 (*p* = 0.11) but not sufficient to change practices [28]. Likewise, a phase 3 trial comparing raltitrexed to 5-FU in stage III CC was closed prematurely due to raltitrexed-induced overmortality and failed to demonstrate non-inferiority of raltitrexed on 5-FU [29].

Thus, the only regimens validated in stage III CC are the oxaliplatin-based chemotherapy FOLFOX or CAPOX, when patients are eligible to a doublet treatment, and LV5FU2 or capecitabine without oxaliplatin in the others.

Compared to FOLFOX, the CAPOX regimen has the advantages of not necessarily requiring the insertion of a central venous access device [30] and reducing the number of injections of oxaliplatin (every 3 weeks) which may be more convenient for the patient and cost-effective. However, in a significant proportion of patients, capecitabine may be contraindicated (renal insufficiency) or poorly tolerated (ileostomy, elderly patients) and leads generally to more frequent and severe diarrhea and hand-foot syndrome. In fragile patients, a tailored-dose escalation strategy for capecitabine prescription could be proposed with an initial dose of 2000 mg/m^2^/d increased if well tolerated to the standard dose of 2500mg /m^2^/d for the second cycle [31].

Finally, dihydropyrimidine dehydrogenase (DPD) deficiency, present in approximately 0.3% of the general population in western countries, has to be tested before adjuvant chemotherapy with fluoropyrimidines, to prevent severe toxicity and possible toxic death in deficient patients [32]. DPD phenotype (assessed by dosing uracilemia) should be preferred to genotyping to detect deficient patients. In case of uracilemia greater than or equal to 150 ng/mL (suggestive of a complete deficit in DPD), treatment with fluoropyrimidines is contraindicated, and their dosage should be substantially reduced in case of uracilemia between 16 and 150 ng/mL.

## 4. Duration of Adjuvant Chemotherapy: IDEA Collaboration

Cumulative sensory neuropathy is one of the major toxicities of oxaliplatin-based adjuvant chemotherapy and can affect the long-term quality of life of patients.

Considering adjuvant treatment duration, the international collaboration IDEA [7] is a pooled analysis of six phase 3 randomised trials across 12 countries, including 12934 patients with stage III CC, and comparing 6 months versus 3 months of FOLFOX/CAPOX (investigators’ choice for chemotherapy regimen) with a primary end point of 3-year DFS. The non-inferiority was not confirmed in the whole population (HR: 1.07; 95% confidence interval (CI), 1.00 to 1.15, with the upper limit of the two-sided 95% CI of the HR of 1.12 necessary to prove non-inferiority). However, the results were different depending on treatment regimen and patients’ risk group.

Stage III CC patients can be classified in low- and high-risk subgroups depending on their T and N stage. T1-3N1 patients are considered low-risk, represent approximately 60% of all stage III patients and show a 3-year DFS of 80%. T4 and/or N2 patients are considered high-risk, represent approximately 40% of all stage III patients and show a 3-year DFS of 60%.

In IDEA low-risk patients, CAPOX for 3 months was shown to be non-inferior to CAPOX for 6 months for DFS (HR: 0.85 95%CI 0.71–1.01), as in high-risk patients, 3 months of chemotherapy was significantly inferior to 6 months in patients treated by FOLFOX (HR: 1.20, 95%CI 1.07–1.35). FOLFOX for 3 months in low-risk patients or CAPOX for 3 months in high-risk patients were neither inferior or superior to 6 months of treatment with the same regimen. 

As expected, the tolerance of chemotherapy was broadly better in the 3-month arm, with a major decrease in patients experiencing grade 2 or higher neurotoxicity, 16.6% with FOLFOX and 14.2% with CAPOX for 3 months as compared to 47.7% with FOLFOX and 44.9% with CAPOX for 6 months. 

Of note, across individual studies, two studies included a higher proportion of T4 tumors (29% versus 12–18% for the others), median follow-up was variable (between 35 and 62 months) and more importantly, the prescription rate for CAPOX was very different, ranging from 0% in the C80702 US trial to 75% in the ACHIEVE Japanese trial.

The updated 5-year IDEA results presented at the ASCO 2020 congress showed for DFS a HR of 0.98 (95%CI 0.88–1.08) in the CAPOX arm between 3 months and 6 months of treatment, while in the FOLFOX arm, DFS was significantly longer with 6 months of treatment (HR 1.16, 95%CI 1.07–1.26). The 5-year OS was not significantly different between 3 months (82.4%) and 6 months of treatment (82.8%) in the whole population (HR:1.02, 95%CI 0.95–1.11). Interestingly, for high-risk stage III patients treated with CAPOX, the absolute difference in 5-year OS rate between 3 and 6 months was only 1% (71.4% versus 72.4%, respectively) with an HR of 1.02 (95%CI 0.89–1.20), while for those treated with FOLFOX, the absolute difference in 5-year OS rate was 2.8% between the two treatment durations (72.5% versus 75.3%) with a HR of 1.12 (95%CI 0.98–1.27) [33].

**Figure 1:**
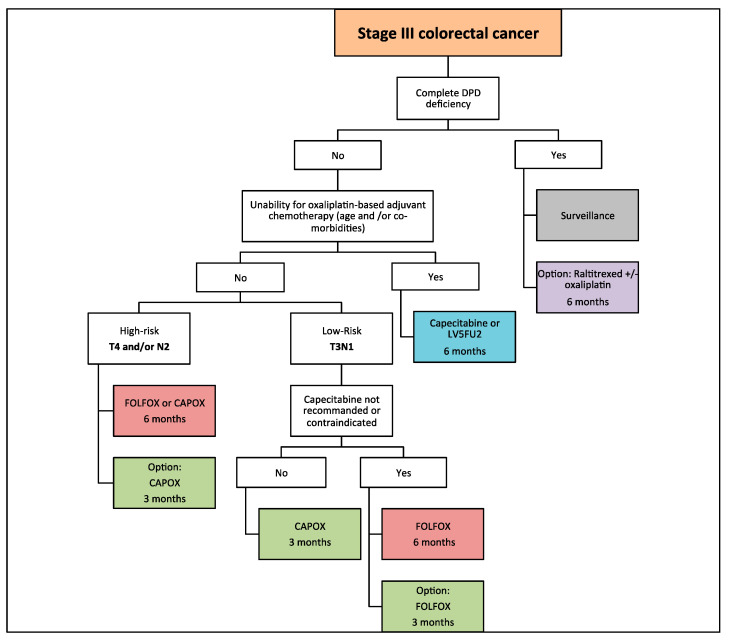
Algorithm for adjuvant chemotherapy in stage III colorectal cancer. DPD: dihydropyrimidine dehydrogenase; LV5FU2: leucovorin 5-fluorouracil.

In conclusion, the primary objective (3-year DFS) and the two secondary objectives (5-year DFS and OS) show minor differences for the majority of patients with stage III CC with 3 months of oxaliplatin-based adjuvant chemotherapy. For low-risk stage III CC, CAPOX for 3 months is the preferred choice and if the patient is not eligible for capecitabine (renal insufficiency, elderly patients, patient whishes), FOLFOX for 3 months can reasonably be proposed because of minor survival differences. For high-risk stage III CC, since 5-year updated results of IDEA show minor differences for OS, CAPOX for 3 months can be proposed, however if the FOLFOX regimen is chosen, it should be prescribed for 6 months (Figure 1).

It is difficult to draw clear conclusions about the difference in efficacy between CAPOX and FOLFOX, because these two regimens were not randomized and they may still have been prescribed to different patient populations. However, it is also possible that capecitabine is superior to LV5FU2 due to its administration schedule (continuous administration 2 weeks/3) which may be more effective on micrometastatic disease, or to an increased efficacy of oxaliplatin administered every 3 weeks to a higher dose (130 mg/m^3^ instead of 85 mg/m^2^ every 2 weeks).

The various phase 3 adjuvant therapeutic trials in stage III CC are listed in Table 1.

## 5. Adjuvant Chemotherapy in Elderly Patients

There are few specific data on adjuvant chemotherapy in patients over 80 years old, who are rarely included in adjuvant therapeutic trials. In addition, the few elderly patients included in these trials are probably highly selected. Compared to patients under 70 years old, elderly patients over 70 years old have a comparable benefit and safety profile of adjuvant fluorouracil-based chemotherapy compared to surgery alone [34,35]. Addition of oxaliplatin did not appear to provide any benefit or minimal benefit in patients over 70–75 years of age in analyses of datasets from the MOSAIC and NSABP C07 trials [18], a population-based study [36] and the ACCENT database [37]. However, the pooled analysis of NSABP C-08, XELOXA, X-ACT, and AVANT trials, including only stage III CC, has shown that FOLFOX or CAPOX compared to LV/5FU led to a survival benefit for all age groups, with a HR at 0.77 (*p* = 0.014) for the subgroup of patients aged over 70 years [38]. 

Knowing that the median age of CC is over 70 years in many countries [1], adjuvant therapeutic trials dedicated to patients over 75–80 years old would be relevant. Some trials are ongoing, such as the PRODIGE34-ADAGE trial testing LV5FU2 versus no chemotherapy in frail patients and versus FOLFOX in fit patients over 70 with stage III CC [39].

In all cases, to decide on an adjuvant chemotherapy in an elderly patient, it is necessary to take into account his co-morbidities, his presumed life expectancy, his performance status, his expectations, and in difficult situations to ask for an oncogeriatric evaluation, to balance well the benefit/risk ratio for each individual patient in multidisciplinary meetings.

## 6. Neo-Adjuvant Chemotherapy for Locally Advanced Colon Cancer

Given the nice results of pre-operative treatments in gastric, esophageal and rectal cancers, neoadjuvant chemotherapy has been explored in colon cancer patients. The PRODIGE 22 phase 2 trial initially showed that neo-adjuvant chemotherapy by FOLFOX (4 cycles) for patients with locally advanced CC (T3/T4 and/or N2 on the initial CT scan evaluation) was well tolerated, with no increase in surgical morbidity and interesting downstaging rates but without significant association with a major histological response (TRG1), compared to patients without neo-adjuvant treatment [40]. The larger phase 3 FOxTROT trial (*n* = 1050 patients) showed the same results in these situations with three cycles of neo-adjuvant FOLFOX, with no significant difference but a strong tendency to decrease the 2-year recurrence rate, which was the primary endpoint of the study, for patients treated with neo-adjuvant FOLFOX, compared to those treated with upfront surgery (13.6% versus 17.2%, respectively, HR: 0.75, *p* = 0.08) [41]. However, for these two trials, the baseline pre-therapeutic CT scan selected in 25 to 30% of cases low-risk CC patients theoretically not requiring chemotherapy, underlining the need for improving pre-treatment disease assessment in this setting. Adding anti-EGFR therapy to neo-adjuvant FOLFOX in patients with RAS wild-type tumors did not provide any benefit in both studies [40,41].

## 7. Biomarkers and Efficacy of Adjuvant Chemotherapy 

Many prognostic biomarkers have been described in stage III CC, but none of them are predictive of adjuvant chemotherapy efficacy and therefore used in current clinical practice. We will review here the various biomarkers described in stage III CC with their possible association with the efficacy of adjuvant chemotherapy. 

A total of 9 to 10% of stage III CCs have a microsatellite instability (MSI) phenotype [42,43,44,45,46,47]. Though MSI bears sufficiently good prognostic information in stage II CC to avoid adjuvant chemotherapy in MSI stage II patients [48], the prognostic value of MSI status is less clear in stage III CC. MSI tumors have a higher frequency of BRAF mutations (for sporadic forms by methylation of hMLH1) which confers poor prognosis [42], thus the best prognostic group are the MSI and BRAF wild-type tumors. MSI tumors are associated with a longer recurrence-free interval (RFI) but showed a poorer survival after recurrence (SAR), possibly due to BRAF-mutated tumors [42,43]. A recent report showed that MSI seems to be of good prognosis in low-risk patients but not in high-risk stage III CC patients [49]. As for stage II tumors, adjuvant chemotherapy with 5FU alone appears to be ineffective in stage III CC in several studies [44,45], whereas the study of Sinicrope et al. showed a benefit of 5FU adjuvant chemotherapy (without oxaliplatin or irinotecan) compared to surgery alone in MSI Lynch patients but not is sporadic cases [46]. Two retrospective studies showed improved DFS with oxaliplatin-based adjuvant chemotherapy as compared to patients treated with 5FU alone [50,51] and the 10-year updated data from the MOSAIC trial confirmed a trend in favor of improved DFS and OS in patients treated with FOLFOX compared to those treated with 5FU alone [5]. 

KRAS mutation (15–35% of localised CC), BRAF V600E mutation (10% of patients with localised CC) and CIMP phenotype (18% of CC) have been all shown in the last decade to be poor prognostic molecular factors with decreased DFS, SAR and OS in many publications [5,47,52,53,54,55]. No significant interactions were seen between BRAF mutation and oxaliplatin treatment in both the MOSAIC and NSABP C-07 trials [5,42]. In the study of Shiovitz et al., CIMP+ patients treated with FOLFIRI trended to have a higher OS compared to those treated with 5FU, unlike CIMP- patients, and this effect was even more pronounced in CIMP+ and MSS patients [56]. We observed in a PETACC-8 trial post-hoc analysis, a non-significant trend towards a detrimental effect of cetuximab in patients with CIMP+ tumors for OS, DFS, and SAR [55].

PIK3CA mutation is present in about 17% [57] of CC and seems to be associated with better survivals for patients treated with aspirin in addition to chemotherapy [58]. In a post-hoc analysis of the VICTOR trial that failed to show efficacy of rofecoxib for mutated PIK3CA and PIK3CA wild type stage II–III tumors, the recurrence rate was lower in aspirin-treated patients in the PIK3CA mutated tumor subgroup, supporting the prospective investigation of adjuvant aspirin in PIK3CA-mutant patients [57]. A French randomised phase III trial (PRODIGE 50-ASPIK) comparing aspirin versus placebo in stage III or high-risk stage II CC is ongoing [59].

Mutational analysis by Next-Generation Sequencing (NGS) in localized CC is not recommended, because it does not indeed modify currently the therapeutic management, however this technique being more easily accessible, can provide prognostic information and perhaps in the future will guide the treatment.

Supervised gene expression signatures in localised CC have been developed to predict the risk of recurrence from the expression of several genes involved in key biological oncogenesis pathways. The most studied ones are: 12-gene Oncotype DX Colon Cancer Recurrence Score [60,61,62], ColoPrint [63,64], Veridex [63,65,66], GeneFx Colon [63,67], ColoGuideEx [68] and 6 microRNA signature [69]. The patients in high-risk groups according to these different gene expression signatures had a significantly higher recurrence rate, independently of classical prognostic factors.

The 12-gene Oncotype DX Colon Cancer Recurrence Score (RS) has been validated in two adjuvant trials including stage III CC: NSABP-C07 (FOLFOX versus 5FU in stage II–III CC) [62] and PETACC-3 (FOLFIRI versus 5-FU in stage II/III CC) [63] trials. In NSABP-C07, there was no interaction between RS and treatment arm but there was a trend toward greater efficacy of oxaliplatin in the high-risk group [62]. However, the level of prognostication currently allowed by these transcriptomic tests is not clinically relevant enough to integrate them in our daily practice. 

Guinney et al. defined four consensus molecular subtypes (CMS) in stage I–IV CC, based on unsupervised classification of whole genome data, with distinguishing features: CMS1 to CMS4 [70]. In the post-hoc analyses from the PETACC-3 and PETACC-8 trials, the worst prognostic group was patients with CMS4 tumors for RFS and OS in multivariate analyses, while the patients with CMS2 tumors had the best SAR; the shortest SAR was seen in patients with CMS1 tumors, consistently with studies that have shown that patients with MSI and BRAF-mutated tumors have a poor prognosis when they relapse [47]. In a post-hoc analysis of the PETACC-8 trial, a deleterious effect of cetuximab was observed in the CMS1 subtype [71]. In addition, Laurent-Puig et al. observed from the same trial that in fact, at least 50% of the tumors simultaneously exhibit characteristics of 2 CMS subtypes, strongly suggesting the existence of intra-tumor heterogeneity [72]. Moreover, the prognosis of the tumors was significantly different according to their heterogeneity [72].

In immunohistochemistry, loss of CDX2 or SMAD4 expression is found in about 5% and 13%, respectively, and are both associated with a poor prognosis [73,74,75,76]. Dalerba et al. showed in a cohort of 1228 patients with stage III CC that adjuvant chemotherapy increased DFS compared to surgery alone, but above all that the benefit of adjuvant chemotherapy was greater in CDX2-negative patients compared to CDX2-positive patients [73]. By contrast, several studies have shown a chemo-resistance to 5-FU for CC with loss of SMAD4 expression [74,75], probably favoured by activation of the Akt pathway. In a post-hoc analysis of the PETACC-3 trial (FOLFIRI versus 5FU in stage II/III CC), the addition of irinotecan seemed to be deleterious for high-risk tumors with loss of SMAD4 expression and conversely might be of higher benefit in patients with retained SMAD4 expression [76].

The Immunoscore^®^ (total tumour-infiltrating T-cell counts and cytotoxic tumour-infiltrating T-cells counts) has been validated in the study of Pagès et al., including 2681 patients with stage I–III CRC from 13 countries, with high Immunoscore^®^ (27%) associated with MSI tumors and the lowest risk of recurrence at 5 years (*p* < 0.0001), independently of prognostic factors (apart from carcinoembryonic antigen (CEA) level and RAS/BRAF mutational status) [77]. The relative contribution of the Immunoscore^®^ to predict survival was better than the known prognostic factors: N stage, T stage, differentiation, VELIPI, sex, and MSI status [77]. The results of Immunoscore^®^ in the IDEA-France trial (including 1062 patients), recently published, have confirmed the prognostic value of Immunoscore^®^ in stage III CC and a beneficial effect of 6 months of FOLFOX/CAPOX in high or intermediate Immunoscore^®^ patients, independently of high-risk/low-risk group (T4/N2 or T1-3/N1), in contrast to patients with a low Immunoscore, for whom 6 months of treatment did not seem to improve patient outcomes [78]. A recent work using artificial intelligence showed that a predictive nomogram based on immune infiltrates and clinical variables identified a group of patients with less than 10% relapse risk and a group with a 50% relapse risk in stage III patients [79]. These findings suggest that machine learning software can assist physicians and pathologists to better define patients’ prognosis.

Finally, probably the most interesting and promising biomarker for minimal residual disease after cancer surgery in stage III CC is the monitoring of circulating tumor DNA (ctDNA). The detection of postoperative ctDNA in plasma of patients with stage I–III CC is highly prognostic for DFS and OS in numerous studies [80,81,82,83]. Reinert et al. recently showed in a prospective cohort of 125 stage I–III CRC patients that longitudinal ctDNA analysis identified 87.5% of relapses, postoperative positive ctDNA was strongly associated with recurrence (HR, 7.2; 95% CI, 2.7–19.0; *P* < .001) and all patients who were ctDNA positive after adjuvant chemotherapy (*n* = 7) experienced relapse [84]. In the patients treated with adjuvant chemotherapy, three of the 10 ctDNA-positive patients were cleared by adjuvant chemotherapy [84]. In the same way, the Australian study by Tie et al. observed in 196 patients with stage III CC that ctDNA was detectable in 17% of patients after the end of adjuvant chemotherapy with 30% of 3-year RFI, compared to 77% of 3-year RFI when ctDNA was undetectable [83]. For the predictive value of ctDNA of the efficacy of chemotherapy according to its duration, from the IDEA France trial (*n* = 805 patients), 13% of all stage III patients were ctDNA positive after surgery and before adjuvant chemotherapy, with more frequently poorly differentiated and perforated tumors [85]. Positive ctDNA was an independent prognostic factor, with 2-year DFS at 64% in ctDNA-positive versus 82% ctDNA-negative patients (adjusted HR: 1.85, *p* < 0.001). The duration of 3 months of chemotherapy seemed associated with a particularly poor outcome in positive ctDNA patients in both high- and low-risk stage III patients [85]. 

## 8. Perspectives for (Neo)Adjuvant Chemotherapy in Stage III Colon Cancer

The molecular characterization of CC during the last decade allowed splitting this apparent homogenous disease into several entities. Roughly, CC can be divided in three major major molecular phenotypes according to genetic and epigenetic alterations of tumor cells: chromosomal instability (70%), MSI (12%) and hypermethylator phenotype (18%). Among these three entities, specific molecular profiles would be interesting to better target for the adjuvant treatment of stage III CC, namely: MMR status, RAS, BRAF, PI3KCA, HER2 mutational profile.

Thus, immune checkpoint inhibitors have shown very promising results for MSI CC in the metastatic setting [86,87], but also showed impressive results among 20 patients with MSI localised CC treated with neo-adjuvant immunotherapy comibining nivolumab + ipilumumab. Among these 20 patients, 12 patients had a pathological complete response [88]. Two trials are currently testing anti-PDL1 monoclonal antibodies postoperatively in stage III CC patients (ATOMIC and POLEM) [89,90].

We will know from the results of the PRODIGE 50-ASPIK trial whether aspirin combined with adjuvant chemotherapy decreases the risk of recurrence in patients with a stage III or high-risk stage II PIK3CA-mutated tumors.

There are also promising results in much rarer molecular subtypes, such as the combination of anti-BRAF, anti-EGFR and anti-MEK (encorafenib, cetuximab, and binimetinib) for metastatic mutated BRAF V600E CC [91], dual HER2 inhibition (pertuzumab plus trastuzumab) in metastatic HER2 mutated or amplified CC [92], larotrectinib for TRK fusion-positive tumors [93] and AMG 510 for KRAS G12C-mutated tumors [94]. These are also interesting tracks to explore in localized CC in the (neo) adjuvant setting. 

For patients with stage III CC at high-risk of recurrence (T4 and/or N2), intensification of adjuvant chemotherapy with the triplet FOLFIRINOX should be of interest, as studied in the IROCAS ongoing study [95], considering that a better 3-year metastases-free survival (78.8% versus 71.7% respectively, *p* = 0.017) was observed in patients treated with FOLFIRINOX before radio-chemotherapy in the PRODIGE 23 trial dedicated to locally advanced rectal cancers [96]. 

Finally, due to its high predictive value for disease recurrence, monitoring ctDNA post-operatively and at the end of adjuvant chemotherapy could greatly guide our therapeutic strategies for localized CC in the future. The Circulate Europe and Circulate–IDEA research groups are currently exploring how to best treat patients with stage III CC on decisions based on ctDNA assessment [97]. 

**Figure 2:**
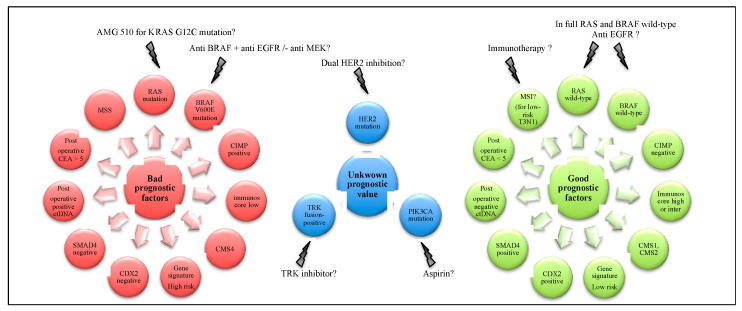
Prognostic markers in stage III colorectal cancer and potential targets for future treatments. Lightnings: potential targeted therapies for molecular subgroups. MSS: microsatellite stable; MSI: microsatellite instability; CEA: carcinoembryonic antigen; ctDNA: circulating tumor DNA; CIMP: CpG island methylator phenotype; HER 2: human epidermal growth factor receptor 2; EGFR: epidermal growth factor receptor; CMS: consensus molecular subtype.

Figure 2 shows the prognostic markers in stage III CC and the potential targets for future treatments.

## 9. Conclusions

For current clinical practice, adjuvant chemotherapy with CAPOX for 3 months may ultimately be sufficient for the vast majority of patients with stage III CC without increased risk of recurrence or death compared to 6 months of CAPOX, in view of the updated DFS and OS results of the IDEA study. For patients not eligible for CAPOX (contraindications, patient preferences), FOLFOX for 3 months is an alternative for low-risk stage III (T3N1) CC but the duration of treatment should remain at 6 months for high-risk stage III (T4 and/or N2) CC. If the patient is too fragile to receive oxaliplatin-based chemotherapy, monotherapy by a fluoropyrimidine for 6 months is an alternative.

In all cases, oxaliplatin must be stopped as soon as the sensory neuropathy has a grade > 1 to avoid long-term sensitive neurotoxicity which may affect the quality of life of patients.

The decision of adjuvant chemotherapy should be done after discussion with the patient about the risks of recurrence, the expected benefit and the possible toxicities of the treatment, and take into account his life expectancy related to possible co-morbidities.

To move forward, it is necessary to be able to better predict individually for each patient the risk of recurrence and the need for adjuvant chemotherapy together with the need of new treatment approaches for specific subgroups. However, we have dramatically improved our knowledge in prognostic markers for resectable CC in the past 15 years. We are currently facing a multitude of individual markers with their own prognostic weight rather than an integrated nomogram that really allows us to perform an accurate and personalized prognostic assessment for each individual patient that may guide our therapeutic choices and clinical trials. In the future, we may be helped by artificial intelligence and machine learning to reach this goal, and ctDNA is a promising tool to stratify patients for specific clinical trials dedicated to treatment intensification and de-escalation. 

## Figures and Tables

**Table 1:** Phase 3 adjuvant therapeutic trials in stage III colorectal cancer. DFS: disease-free survival; OS: overall survival.

First Author	Study Name	*N*	Year	Stage	Chemotherapy	Duration	DFS				OS			
							3–year DFS	5–year DFS	HR	*p*	3–year OS	5–year OS	HR	*p*
IMPACT [11]	IMPACT	652	1982–1989	III	FULV	6 months	62%		0.55	< 10^−4^	76%		0.71	0.02
					Surgery alone		44%				64%			
Francini et al. [13]		118	1985–1990	III	FULV	12 months						69%		0.0025
					Surgery alone							43%		
O Connell et al. [14]		317	1988–1989	II–III	FULV	6 months		74%						0.01
					Surgery alone			58%						
André et al. [15]		905	1996–1999	II–III	LV5FU2	6/9 months	73%		1.04	0.7	86%		1.26	0.18
					FULV	6/9 months	72%				88%			
Twelves et al. [16]	X-ACT	1987	1998–2001	III	Capecitabine	6 months	64%		0.87	0.12	81%		0.84	0.07
					FULV	6 months	61%				78%			
André et al. [4]	MOSAIC	2246	1998–2001	II–III	FOLFOX	6 months	78%		0.76	0.002	88%		0.90	
					LV5FU2	6 months	73%				87%			
Ychou et al. [23]	FNCLCC Accord02 /FFCD9802	400	1998–2002	III	FOLFIRI	6 months	51%		1.19	0.22		61%	1.20	0.26
					LV5FU2	6 months	60%					67%		
Popov et al. [29]	PETACC-1	1921	1999	III	Raltitrexed	6 months						62%	1.04	
					FU/LV	6 months						62%		
Saltz et al. [22]	CALGB 89803	1264	1999–2001	III	CPT-11 + FU/LV	8 months	66%	59%		0.85	80%	68%		0.74
					FU/LV	8 months	69%	61%			81%	71%		
Kuebler et al. [17] Yothers et al. [18]	NSABP C-07	2409	2000–2002	II–III	FLOX	6 months		69%	0.82	0.002		80%	0.88	0.08
					FULV	6 months		64%				78%		0.09
Van Cutsem et al. [24]	PETACC-3	2094	2000–2002	III	FOLFIRI	6 months		57%	0.9	0.1		74%		
					LV5FU2	6 months		54%				71%		
Haller et al. [19]	XELOXA	1886	2003–2004	III	XELOX	6 months		66%	0.80	0.004		78%	0.87	0.15
					FU/FA	6 months		60%				74%		
Allegra et al. [20]	NSABP C-08	2672	2004–2006	II–III	FOLFOX + bevacizumab	FOLFOX 6 months Bevacizumab 1 year	77%		0.89	0.15		82%	0.95	0.56
					FOLFOX	6 months	75%					81%		
De Gramont et al. [26]	AVANT	2867	2004–2007	III	FOLFOX +Bevacizumab	6 months 1 year	73%		1.17	0.07		81%	1.27	0.02
					CAPOX + Bevacizumab	6 months 1 year	75%		1.07	0.44		82%	1.15	0.21
					FOLFOX		76%					85%		
Alberts et al. [21]	NCCTG NO147	1863 (KRAS WT)	2004–2009	III	FOLFOX + cetuximab	6 months	71%		1.21	0.08	86%		1.25	0.15
					FOLFOX	6 months	75%				87%			
Taieb et al. [25]	PETACC-8	1602 (KRAS WT)	2005–2009	III	FOLFOX + cetuximab	6 months	75%		1.05	0.66	88%		1.09	0.56
					FOLFOX	6 months	78%				90%			
Kerr et al. [27]	QUASAR,2	1941	2005–2010	II–III	Capecitabine +Bevacizumab	6 months 1 year	75%		1.06	0.54	87%		1.11	0.33
					Capecitabine	6 months	78%				89%			
Grothey et al. [7] Sobrero et al. [33]	IDEA	12834	2007–2015	III	FOLFOX/CAPOX	3 months	75%		1.07	0.11		82%	1.02	0.64
					FOLFOX/CAPOX	6 months	75%					83%		
